# Where is students’ research in evidence-informed decision-making in health? Assessing productivity and use of postgraduate students’ research in low- and middle-income countries: a systematic review

**DOI:** 10.1186/s12961-017-0169-9

**Published:** 2017-03-09

**Authors:** E. A. Obuku, J. N. Lavis, A. Kinengyere, D. K. Mafigiri, F. Sengooba, C. Karamagi, N. K. Sewankambo

**Affiliations:** 1 Clinical Epidemiology Unit, Department of Medicine, School of Medicine, College of Health Sciences, Makerere University, P.O. Box 7072, Kampala, Uganda; 2 McMaster Health Forum, Centre for Health Economics and Policy Analysis, Department of Clinical Epidemiology and Biostatistics and Department of Political Science, McMaster University, Hamilton, Canada; 3 Department of Health Policy and Planning, School of Public Health, College of Health Sciences, Makerere University, Kampala, Uganda; 4 Department of Social Work and Social Administration, College of Humanities and Social Sciences, Makerere University, Kampala, Uganda; 5 Sir Albert Cook Library, College of Health Sciences, Makerere University, Kampala, Uganda; 6 Department of Global Health and Population, Harvard School of Public Health, Harvard University, Massachusetts, United States of America; 7 Center for Social Science Research on AIDS, Department of Anthropology, College of Arts and Sciences, Case Western Reserve University, Cleveland, OH United States of America; 8 Faculty of Epidemiology and Population Health, London School of Hygiene and Tropical Medicine, London, United Kingdom

**Keywords:** Student, Productivity, Publication, Citation, Bibliometrics, Knowledge translation

## Abstract

**Background:**

Investing in research that is not accessible or used is a waste of resources and an injustice to human subject participants. Post-graduate students’ research in institutions of higher learning involves considerable time, effort and money, warranting evaluation of the return on investment. Although individual studies addressing research productivity of post-graduate students are available, a synthesis of these results in low-income settings has not been undertaken. Our first aim is to identify the types of approaches that increase productivity and those that increase the application of medical post-graduate students’ research and to assess their effectiveness. Our second aim is to assess the determinants of post-graduate students’ research productivity.

**Methods:**

We propose a two-stage systematic review. We will electronically search for published and grey literature in PubMed/MEDLINE and the ERIC databases, as well as contact authors, research administration units of universities, and other key informants as appropriate. In stage one, we will map the nature of the evidence available using a knowledge translation framework adapted from existing literature. We will perform duplicate screening and selection of articles, data abstraction, and risk of bias assessments for included primary studies as described in the Cochrane handbook for systematic reviews. Our primary outcome is publication output as a measure of research productivity, whilst we defined research use as citations in peer-reviewed journals or policy-related documents as our secondary outcome. In stage two, we will perform a structured narrative synthesis of the findings and advance to quantitative meta-analysis if the number of studies are adequate and their heterogeneity is low. Adapting the Grading, Recommendations, Assessment, Development and Evaluation (GRADE) approach, we will assess the overall quality of evidence for effects, and report our results in line with the Preferred Reporting Items for Systematic Reviews and Meta-Analyses (PRISMA) statement.

**Discussion:**

We will share our findings with universities, other training institutions, civil society, funders as well as government departments in charge of education and health particularly in low- and middle-income countries.

## Background

Throughout the world, in universities and other institutions of higher learning, students complete research projects as a prerequisite for obtaining their academic awards. Consequently, resources are invested in terms of teaching and research hours, grant awards, student’s learning efforts and voluntary involvement of research participants with the expectation that, ultimately, these research projects will create new knowledge or influence health policies [1] or published research reports will be available for users like systematic reviewers. Otherwise, investment in students’ research (herein defined as research conducted by post-graduate students in pursuit of an academic qualification such as masters or doctorate degrees) could be viewed as a waste of resources [2] and an injustice to the patients, collaborators, funders and the scientific community at large [3, 4]. What actually happens to this pool of knowledge resulting from post-graduate students’ research projects is a subject for further study.

Although individual studies addressing research productivity of post-graduate students are available, a synthesis of these results has not been undertaken except for medical and surgical residency programmes in the United States of Amerca and Canada, where Stevenson et al. [5] found that protected time, research curricula, or specialised research tracks increased participation in scholarly activity (presentations or publications) of clinical residents. Post-graduate students operate in a unique academic environment from established university researchers characterised by obligatory theses, encounters with supervisors and a limited period within which to graduate from the programme. Studies that document the pathway of students’ research generally show that a substantial proportion of this work ends up on shelves as grey literature and thus underutilised [6–8]. While there is some research evidence proposing approaches that may increase research productivity of post-graduate students, for example mentorship and twinning programmes [9] or mentorship for manuscript writing [10], evidence is scarce about approaches for increasing the application of students’ research beyond publication, a concept known as knowledge translation.

Our first aim is to identify the types and assess the effectiveness of approaches that increase productivity (as measured by publication output) and approaches that increase the application of medical post-graduate students’ research (as measured by citations in peer-reviewed journals or policy-related documents). Our second aim is to assess the determinants of post-graduate students’ research productivity.

## Methods

### Design and justification

We propose a multi-stage systematic review of effects [11]. Systematic review methodology, compared to traditional reviews, has demonstrated a lower risk of bias in information synthesis, thereby increasing confidence in the evidence generated [12–14]. Our multi-stage approach is necessary as there has not been a similar review on this subject and the literature remains unexplored. We will develop a conceptual map of the type and quantity of published and grey literature about post-graduate students’ research projects before proceeding to synthesising our findings on the types of approaches to increasing productivity, application and determinants of productivity.

### Description of the approaches for increasing productivity and use of research

**Fig. 1:**
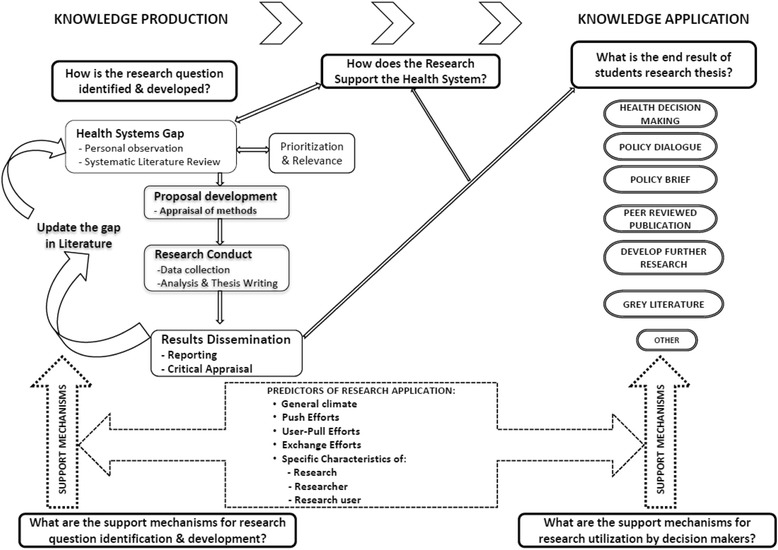
Framework for the systematic review of literature of strategies for knowledge translation of graduate students’ research in institutions of higher learning

Knowledge translation (KT) has been defined as “*… a dynamic and iterative process that includes the identification, synthesis, dissemination, exchange and ethically sound application of knowledge to improve health, provide more effective health services and products and strengthen the healthcare system…*” [15]. Production of knowledge through research is a key component of KT that is followed by its application to address prevailing health or health systems problems [16]. We adapted our KT framework (Fig. 1) from that of Lavis et al. [17], which assesses country level efforts to link research to action. Here, the process of generating research is described in the left column of the framework headed ‘knowledge production’, whilst the use of research falls under ‘knowledge application’. In between are support mechanisms including ‘push efforts’ (our focus), which denotes a cluster of activities to feed decision-makers, both at local level, such as the hospital management teams, district health teams and ministries of health, and at the international level such as WHO, with appropriately packaged actionable messages [17]. ‘Pull efforts’ are characterised by decision-makers demanding for this information [17], which we will not attend to in this review.

We define a student as a post-graduate learner or scholar who is registered in a health training or research institution. Such a student would have undertaken research activities usually leading to a master’s or doctorate degree or their equivalent. In some instances students undergoing fellowship training in a specific health field are not expected to obtain a specific diploma or degree award [18]. During post-graduate training, students are provided with academic support mechanisms and are expected to complete an independent research project as part of their studies. Support mechanisms that could increase productivity include priority setting exercises; research supervision or mentorship by a more senior academic; protected time for research, obligatory requirement to publish, research grant awards; and seminars for proposal development, peer review, data analysis and manuscript writing. Approaches that specifically target increasing the use of research include training sessions in research dissemination sessions, skills building platforms for completing policy briefs or policy dialogues, or actively engaging decision-makers, all of which constitute ‘push efforts’.

### Systematic review procedures

We have written this protocol paying attention to signaling questions in the following guidance tools for the quantitative aspects of this systematic review, that is, the systematic review of effects: Methodological Expectations of Cochrane Intervention Reviews (MECIR) by the Cochrane Collaboration [19]; Preferred Reporting Items for Systematic Reviews and Meta-analyses Protocols (PRISMA-P) [20]; and the Assessing Methodological Quality of Systematic Reviews (AMSTAR) tool for placing confidence in reviews [21].

### Search strategy

#### Electronic search

Our initial screening of an electronic search of 3460 titles and abstracts in PubMed/Medline yielded 176 (5.1%) as potentially eligible (Table 1). This search explores various combinations of the following keywords covering the elements of “PICOS” as follows: (1) Population – student, trainee, graduate, post-graduate, nursing, pharmacy, medicine, dentistry, resident, public health; (2) intervention or exposure – doctorate, post-doctorate, fellowship, grant, scholarship, research award, masters, mentorship, supervisor, PhD, research, training, workshop; (3) comparator – we will not employ specific terms in the electronic search for the comparator as these are already captured in the terms describing the intervention; (4) outputs/outcomes – thesis, dissertation, abstract, publication, journal article, technical report, book chapter, conference presentation, policy brief, policy dialogue, decision or policymaking; (5) study design – in order to minimise the risk of an empty review, we will not enter specific terms for the study design as we intend to use all evidence types to describe the available range of interventions. The full search string is given in Supplement 1.

**Table 1:** Feasibility of yield of literature of pilot electronic search strategy for post-graduate student’s research

Search number (Data base)	Search terms (and date)	Number of hits^a^ (Relevant)
#7 (PubMed)	(((((((Medicine[tiab] OR Nursing[tiab] OR Dentistry[tiab] OR Pharmacy[tiab] OR “Public health”))) AND ((Degree*[tiab] OR Doctor*[tiab] OR Post-doc*[tiab] OR PhD[tiab] OR Master*[tiab] OR Fellow*[tiab] OR Residen*[tiab] OR Student*[tiab] OR Trainee*[tiab] OR Graduate*[tiab] OR Post-grad*[tiab])))) AND ((Mentor*[tiab] OR Grantee [tiab] OR Fund*[tiab] OR Supervis*[tiab] OR Workshop*[tiab] OR Seminar*[tiab] OR Conference*[tiab] OR “Manuscript-writing”[tiab] OR "Scientific-writing”[tiab] OR "Academic-writing”[tiab] OR "Scholarly-writing”[tiab] OR “Grants-writing”[tiab] OR “Capacity building”[tiab] OR Research[tiab])))) AND ((Abstract*[tiab] OR Thesis[tiab] OR Theses[tiab] OR Dissertation*[tiab] OR publication*[tiab] OR “Poster session” OR “Poster presentation” [tiab] OR “Book chapter” [tiab] OR “Technical report”[tiab] OR “Policy brief”[tiab] OR “Policy dialogue” [tiab] OR “Evidence informed policy”[tiab] OR “Evidence based policy”[tiab] OR “Evidence informed health policy”[tiab] OR “Evidence based health policy”[tiab] OR “Decision making”[tiab] OR “Policy making”[tiab] OR Dissemination[tiab]))	^a^3460 (^b^176, 5%)

^a^Number of article titles and abstracts as at 21 June 2016

^b^Sorted by relevance and initial screening of titles and abstracts

We will search the following open access electronic databases: PubMed/Medline (http://www.ncbi.nlm.nih.gov/pubmed) and the Education Resource and Information Centre (ERIC, www.eric.ed.gov) without introducing restrictions by language or time period. Instead, where articles exist in languages other than English, we will use Google translator (www.translate.google.com) to translate texts into English and then carry out initial screening before proceeding to full text retrieval or exclusion of the study.

#### Additional searches

We will screen the reference lists of relevant publications and contact authors or heads of academic or research departments in target universities as key informants to identify university reports, unpublished or ongoing evaluations about productivity and use of post-graduate students’ research. In order to contact relevant post-graduate institutions, we will use existing contact lists of university medical or public health schools in low- and middle-income countries as per the World Bank country income status [22].

### Selection of studies

#### Data management

Using EndNote software version *X7* (Thomson Reuters, 2015) we will import all identified titles, exclude duplicates, screen and group these into relevant eligibility categories described below. In order to improve transparency of our review process, we will depict the selection of primary studies and reasons for exclusion in a flow chart [23].

#### Minimising bias in study identification and selection

In order to minimise the risk of selection bias in our systematic review conduct, a second reviewer will validate the electronic search in PubMed by performing an independent and duplicate search. Similarly, the second reviewer will screen all articles excluded by the first reviewer. We will carry out independent study selection and data abstraction and resolve any differences by discussion and consensus.

#### Criteria for considering inclusion of studies

We will include published studies and grey literature reporting at least one outcome of interest, as defined in the section of outcome variables. We have chosen to include studies from all countries to facilitate comparisons, increase the relevance and optimise the existing evidence. Hence, we will include all types of study designs to measure effects of these approaches such as controlled trials (randomised or non-randomised), interrupted time-series, controlled before-and-after studies, and cohort studies (prospective or retrospective). Observational designs (cross-sectional and case–control) will be key in estimating the prevalence and determinants of productivity as well as mapping the types of existing approaches.

#### Exclusion criteria for ineligible studies

We will exclude studies about research conducted by bachelor’s degree or undergraduate students’ or established university faculty not identified as post-graduate students; as well as those studies where we are unable to obtain key information to aid eligibility decisions. In many universities throughout the world, research projects and theses are a requirement for post-graduate academic qualifications, which is not necessarily the case for most Bachelors (undergraduate) programmes. Additionally, we will exclude studies about medical degree trainees in systems where medical, pharmacy, nursing or dental schools are considered post-graduate training, similar to the United States and Canada [5].

### Data abstraction

We will adapt the Cochrane Collaboration’s good practice data extraction form for effectiveness studies [24]. This includes administrative, study design and primary outcome data on (1) productivity measured as proportion of publications. Our secondary outcome is (2) use of the research in policy-related documents or processes (systematic reviews or policy briefs or technical reports or policy dialogues) or informing new research as measured by citations. Additional outcomes are described above, in the section under electronic search by the PICOS framework.

### Preliminary results

In order to gauge the viability of our review, we have listed five studies that meet our eligibility criteria from the PubMed search, after initial screening of titles and abstracts (Table 2). These studies were published between 2010 and 2014, and conducted in universities in Africa (Cameroon and Egypt) [25, 26], Europe (Turkey) [27], and Asia (India, Iran) [28, 29]. These studies addressed post-graduate research among students pursuing only masters (n = 3) or Masters and Doctorate degrees (n = 2). The proportion of publications ranged from 12% to 40%, which is our primary outcome as reported by all the five studies.

**Table 2:** Preliminary findings of potentially eligible studies in low- and middle-income countries

Author	Year	Country	Design	Setting/Population	^a^Publication
Dhaliwal [28]	2010	India	Cross-sectional	Masters	30%
Sipahi [27]	2012	Turkey	Cross-sectional	Masters	12%
Munung [25]	2014	Cameroon	Cross-sectional	Masters & Doctoral	14%
Motamed-Jahromi [29]	2014	Iran	Cross-sectional	Masters	40%
Nour-Eldein [26]	2015	Egypt	Cross-sectional	Masters & Doctoral	22%

^a^Proportion of theses that were published as full text articles in peer reviewed journals. This is the primary outcome of the systematic review

### Handling of missing data

We will denote variables that are desired but missing or not reported as ‘NR’, and seek clarification by contacting the authors. We will not employ any statistical methods for handling missing data.

### Risk of bias of assessment of included studies

We will assess for the risk of bias of included studies by adapting the Cochrane risk of bias tool for randomised and non-randomised studies. Random sequence generation, allocation concealment, contamination protection and attrition are the key quality aspects we will assess for in trials [19]. Otherwise, for the observational studies, we will consider the following specific risk of bias aspects: similarity of baseline characteristics, sample size, control group, instrumental variables or potential confounding for all types of observational designs; questionnaire validity and reliability, sampling strategy, response rates for cross-sectional studies; attrition for cohort studies; choice of controls for case–control studies; analysis strategy, namely complete cases, per-protocol, as-treated or intention-to-treat; assessment for regression to the mean for controlled before and after or interrupted time-series designs [30].

### Assessing the overall quality of evidence

We will adopt the Grading Recommendations Assessment, Development and Evaluation (GRADE) approach, which appraises the overall quality of evidence related to selected key outcomes for quantitative studies [31]. Indeed, we acknowledge that GRADE is rarely applied in our area of research; nevertheless, we will apply the concepts to assess the quality of evidence. In the GRADE method, the quality of evidence is rated for each key outcome as ‘High’, ‘Moderate’, ‘Low’, or ‘Very Low’. Observational studies start at low quality and may be upgraded, whilst randomised trials are set at high quality and may be downgraded. GRADE applies the following criteria: risk of bias, inconsistency, imprecision, indirectness, and publication bias when downgrading the quality of evidence whilst the magnitude of effect, dose–response and confounding are considered when upgrading. Using GRADE, we will develop summary of findings tables, and assess the confidence in the effect estimates, and strength of recommendations based on the quality of evidence. Indeed, with low quality or absence of evidence, we will identify areas for further research.

### Synthesis of included studies

We propose a structured approach to synthesise findings of this review. The unit of analysis will be findings from a single primary study. First, we will describe the characteristics of the included primary studies. Using Stata version 14.1 (Stata, College Station, Texas, USA) we will construct forest plots using frequencies and proportions for the quantitative studies. Where there are relative or absolute or change from baseline measures of effect, for example, risk difference, odds ratios, risk ratios or mean change, we will standardise these measures by obtaining frequencies or counts and re-computing the appropriate measure of effect. Contextual issues in different research institutions, countries, post-graduate programme, and years of study are likely to manifest variation, which we will explore visually by inspecting the forest plots and statistically quantifying this using the *I* ^*2*^ statistic and testing for significance using Cochran’s Q. We will combine the results quantitatively if we are satisfied that the level of heterogeneity will not affect the overall interpretation of the effect estimates. We will also investigate for publication bias if there is a reasonable number of included studies (> 10) using multiple methods, visually using a funnel plot, or statistically using Begg’s or Egger’s tests.

### Reporting, dissemination and KT strategy

We will align our reporting to the PRISMA statement [23] and share the full report with relevant stakeholders including universities, civil society, funders, and departments of education and health in low-and middle-income countries, and eventually post it to the Uganda Clearing House for Health Policy and Systems Research (http://www.chs.mak.ac.ug/uch/home).

## Discussion

### Anticipated methodological limitations

We anticipate two main methodological limitations in our review. First, the identification of all the relevant studies or university reports will be limited by grey literature beyond the reach of our review team. We will address this by exploring publication bias, which will inform our interpretation of the findings. Secondly, identifying the outcome of ‘use’ of students’ research in the policy process or decision-making in health remains a challenge and few primary studies are likely to report this. Thus, application or use of studies in decision-making is premised on accessibility through some form of publication either as a policy-related document or a peer reviewed journal article.

## Abbreviations

AMSTAR: measurement tool to assess systematic reviews
ERIC: Education Resources Information Centre
GRADE: Grading of Recommendations Assessment, Development and Evaluation
KT: knowledge translation
MECIR: Methodological Expectations of Cochrane Intervention Reviews
PICOS: Population Intervention Comparison Outcome and Study design
PRISMA: Preferred Reporting Items for Systematic reviews and Meta-Analyses

## References

[1] Kebede D, Zielinski C, Mbondji PE, Sanou I, Kouvividila W, Lusamba-Dikassa PS (2014). Research output of health research institutions and its use in 42 sub-Saharan African countries: results of a questionnaire-based survey. J R Soc Med.

[2] Chalmers I, Glasziou P (2009). Avoidable waste in the production and reporting of research evidence. Lancet.

[3] Chalmers I (2012). For ethical, economic and scientific reasons, health-relevant degree theses must be made publicly accessible. Evid Based Med.

[4] Chalmers I (1990). Underreporting research is scientific misconduct. J Am Med Assoc.

[5] Stevenson MD, Smigielski EM, Naifeh MM, Abramson EL, Todd C, Li ST (2017). Increasing scholarly activity productivity during residency: a systematic review. Acad Med.

[6] Bullen CR, Reeve J (2011). Turning postgraduate students' research into publications: a survey of New Zealand masters in public health students. Asia Pac J Public Health.

[7] Dickersin K, Min YI, Meinert CL (1992). Factors influencing publication of research results. Follow-up of applications submitted to two institutional review boards. JAMA.

[8] Caan W, Cole M (2012). How much doctoral research on clinical topics is published?. Evid Based Med.

[9] Heimburger DC, Carothers CL, Blevins M, Warner TL, Vermund SH (2015). Impact of global health research training on scholarly productivity: The Fogarty International Clinical Research Scholars and Fellows Program. Am J Trop Med Hyg.

[10] Dowling DA, Savrin C, Graham GC (2013). Writing for publication: perspectives of graduate nursing students and doctorally prepared faculty. J Nurs Educ.

[11] Gough D, Thomas J, Oliver S (2012). Clarifying differences between review designs and methods. Syst Rev..

[12] Chalmers I, Altman D (1995). Systematic Reviews.

[13] Greenhalgh T (1997). Papers that summarise other papers (systematic reviews and meta-analyses). BMJ.

[14] Greenhalgh T (1999). How to read a paper. The basics of evidence based medicine.

[15] Canadian Institute for Health Research (2004). Knowledge translation strategy 2004 - 2009: Innovation in action.

[16] Straus SE, Tetroe J, Graham I (2009). Defining knowledge translation. CMAJ.

[17] Lavis JN, Lomas J, Hamid M, Sewankambo NK (2006). Assessing country-level efforts to link research to action. Bull World Health Organ.

[18] Heimburger DC, Carothers CL, Gardner P, Primack A, Warner TL, Vermund SH (2011). Nurturing the global workforce in clinical research: the National Institutes of Health Fogarty International Clinical Scholars and Fellows Program. Am J Trop Med Hyg.

[19] Chandler J, Churchill R, Higgins J, Lasserson T, Tovey D. Methodological standards for the conduct of new Cochrane Intervention Reviews. London. 2013. http://editorial-unit.cochrane.org/mecir. Accessed 27 December 2014.

[20] Moher D, Shamseer L, Clarke M, Ghersi D, Liberati A, Petticrew M (2015). Preferred reporting items for systematic review and meta-analysis protocols (PRISMA-P) 2015 statement. Syst Rev..

[21] Shea BJ, Hamel C, Wells GA, Bouter LM, Kristjansson E, Grimshaw J (2009). AMSTAR is a reliable and valid measurement tool to assess the methodological quality of systematic reviews. J Clin Epidemiol.

[22] The World Bank (2013). New Country Classifications.

[23] Moher D, Liberati A, Tetzlaff J, Altman DG (2009). Preferred reporting items for systematic reviews and meta-analyses: the PRISMA statement. BMJ..

[24] Higgins JPT, Green S. Cochrane Handbook for Systematic Reviews of Interventions Version 5.1.0. Updated March 2011. The Cochrane Collaboration; 2011. http://handbook.cochrane.org/. Accessed 1 Mar 2016.

[25] Munung N, Vidal L, Ouwe-Missi-Oukem-Boyer O (2014). Do students eventually get to publish their research findings? The case of human immunodeficiency virus/acquired immunodeficiency syndrome research in Cameroon. Ann Med Health Sci Res.

[26] Nour-Eldein H, Mansour NM, Abdulmajeed AA (2015). Master's and doctoral theses in family medicine and their publication output, Suez Canal University, Egypt. J Fam Med Prim Care.

[27] Sipahi H, Durusoy R, Ergin I, Hassoy H, Davas A, Karababa A (2012). Publication rates of public health theses in international and national peer-review journals in Turkey. Iran J Public Health.

[28] Dhaliwal U, Singh N, Bhatia A (2010). Masters theses from a university medical college: publication in indexed scientific journals. Indian J Ophthalmol.

[29] Motamed-Jahromi M, Leila DS (2014). Nursing MSc theses: a study of an Iranian college of nursing and midwifery in two decades (1990-2010). Glob J Health Sci.

[30] Vandenbroucke JP, von Elm E, Altman DG, Gøtzsche PC, Mulrow CD, Pocock SJ (2014). Strengthening the Reporting of Observational Studies in Epidemiology (STROBE): explanation and elaboration. Int J Surg.

[31] Guyatt GH, Oxman AD, Vist GE, Kunz R, Falck-Ytter Y, Alonso-Coello P (2008). GRADE: an emerging consensus on rating quality of evidence and strength of recommendations. BMJ.

